# Factors associated with crack-cocaine early initiation: a Brazilian multicenter study

**DOI:** 10.1186/s12889-021-10769-x

**Published:** 2021-04-23

**Authors:** Luciane Ogata Perrenoud, Koki Fernando Oikawa, Anna Virginia Williams, Ronaldo Laranjeira, Benedikt Fischer, John Strang, Marcelo Ribeiro

**Affiliations:** 1 Department of Psychiatry, Federal University of São Paulo (UNIFESP), São Paulo, Brazil, Rua Major Maragliano, 241, SP 04017030 São Paulo, Brazil; 2 Reference Center for Alcohol, Tobacco and Other Drugs (CRATOD), São Paulo State Secretary of Health, Rua Prates, 165, 01121000 São Paulo, Brazil; 3 Department of Statistics, Brazil University, São Paulo, Brazil, Rua Ibipetuba, 130, SP 03127-180 São Paulo, Brazil; 4 National Addiction Centre, Institute of Psychiatry, Psychology and Neuroscience, King’s College London, London, UK, 4, Windsor Walk Denmark Hill, SE5 8AF London, UK; 5 Schools of Population Health and Pharmacy, Faculty of Medical and Health Sciences, University of Auckland, Auckland, New Zealand; 6 Centre for Applied Research in Mental Health and Addiction, Simon Fraser University (SFU Faculty of Health Sciences, 515 W. Hastings Street, Vancouver, BC V6B 5K3), Canada; 7 Department of Psychiatry, University of Toronto, 250 College Street, 8th floor, Toronto, ON. M5T 1R8 Canada

**Keywords:** Crack-cocaine, Parental monitoring, Deviant behavior, Risk and protective factors, Therapeutic communities, Age of onset, Drug use

## Abstract

**Background:**

Crack-cocaine dependence is a serious public health issue, related to several psychiatric and psychosocial problems. Crack-cocaine users are usually embedded in a context of great social vulnerability, often associated with violence, poverty, family conflict and easy and early access to alcohol, tobacco and other drugs.

**Methods:**

This cross-sectional study enrolled a consecutive sample of 577 patients admitted to 20 therapeutic communities located in Southern Brazil, between September 2012 and September 2013. A structured interview assessed life-time exposure to risk and protective factors for drug use, such as parental monitoring in childhood, deviant behaviors and peer pressure.

**Results:**

Presence of family conflict (*p* = 0.002), maltreatment (*p* = 0.016), and deviant behavior prior to age 15 in a bivariate analysis predicted an earlier age of crack-cocaine initiation, whereas adolescents experiencing parental monitoring during adolescence started use later (*p* < 0.001). In the multivariate model, perceptions related to ease of access of illicit drugs (marijuana: *p* = 0.028, 95% CI = − 3.81, − 0.22; crack-cocaine: *p* < 0.001, 95% CI = − 7.40, − 4.90), and deviant behavior (threatening someone with a gun: *p* = 0.028, 95% CI = − 2.57, − 0.14) remained independent predictors of early age of crack-cocaine initiation.

**Conclusions:**

Early onset of crack-cocaine use seems to be associated with exposure to family conflict, easy access to drugs and deviant behavior. Treatment and preventive programs should take these factors into account when designing and implementing community interventions.

**Supplementary Information:**

The online version contains supplementary material available at 10.1186/s12889-021-10769-x.

## Background

Crack-cocaine use, and other forms of smokable cocaine, is a serious public health problem that affects virtually all countries across the Americas and some nations in Central and Eastern Europe [1, 2]. Crack-cocaine users tend to develop severe pattern of dependence in a very short period of time [3–5]. The use of this drug is associated with psychiatric comorbidities, including depression, antisocial personality disorder and suicide attempts [6]. In addition, its use is also associated with sexual risk behaviors and high rates of Human Immunodeficiency Viruses (HIV) and hepatitis B and C viruses’ infections, exceeding those observed in the general population [7]. Evidence indicates elevated mortality rates among crack-cocaine users, well above international standards, homicide being the main cause of death [8, 9].

In Brazil, crack-cocaine users are often part of vulnerable and marginalized groups in society and suffer from severe social and economic disadvantage [10, 11]. Exposure to violence and situations of abuse have also been associated with crack-cocaine use [7].

The first experience with crack-cocaine usually occurs in the transition to adulthood, often following the exposure to alcohol, tobacco, marijuana and snorted cocaine [4, 12]. According to Substance Abuse and Mental Health Services Administration (SAMHSA), crack-cocaine first use generally occurs between the ages of 18 and 25 in the United States [13]. Likewise, a study with homeless people (*n* = 203) from Montreal, Canada, found that the age of onset of cocaine use and its presentations, such as crack-cocaine, happened after 17 years of age [14]. The II Brazilian National Alcohol and Drugs Survey (BNADS) found that the mean age of initiation for cocaine consumption in Brazil was 18.8 years [15]. Another survey with Brazilians adolescents in treatment found that the mean age at first use of crack-cocaine was 13.3 years [4].

The age of onset of drug consumption is influenced by numerous combinations of protective and risk factors, within a system that integrates social environments, relationships, individual characteristics and behavioral patterns [4, 16]. Longitudinal studies with psychoactive substances users have shown that the earlier the age of onset of alcohol and tobacco use the greater the likelihood of developing drug addiction, more severe drug use patterns and more problematic deviant behaviors [14, 16]. Therefore, assessing the risk factors related to the early age of drug use initiation could help to plan and implement preventive strategies [17].

Many risk factors for drug use initiation are part of a complex and multifaceted framework which involves an interplay of genetic, psychological and social factors [18–21]. Individual and personality factors such as curiosity, impulsivity and sensation seeking are common in adolescence [22–24]. In addition, the perception of easy access is strongly related to the risk of starting psychoactive substances use [25]. Use of drugs by parents, family conflict and peer pressure also appear to be associated with future problematic drug use [17, 19, 26, 27]. The Brazilian National Survey of Crack-Cocaine Users [28] found that the main reasons for first crack-cocaine use were curiosity (58.3%), followed by family conflicts (29.2%) and influence of friends (26.7%).

Studies evaluating adolescent alcohol use suggest that parents monitoring their children’s routine and expressing disapproval of drug use are protective factors for both: early onset and binge drinking [21, 29, 30]. In contrast, children with parents who drink or are permissive in relation to drug use appear to have an early onset of substance use, suggesting that early patterns of use may be influenced by social and familial environmental factors [17–20].

More recently, cross-sectional and longitudinal studies, assessing predictors of early initiation of crack-cocaine use, found an association with sociodemographic factors, psychiatric comorbidities and previous use of any drug [4, 31–33]. Other studies investigated specific populations - for example, adolescents in street situations [14], however few multicenter studies focused on crack-cocaine users. To our knowledge, this if the first multicenter study in Brazil assessing risk and protective factors for early onset of crack-cocaine use with patients from Therapeutic Communities (TCs) who identified crack-cocaine as the substance that made them seek treatment.

## Methods

### Setting

**Fig. 1:**
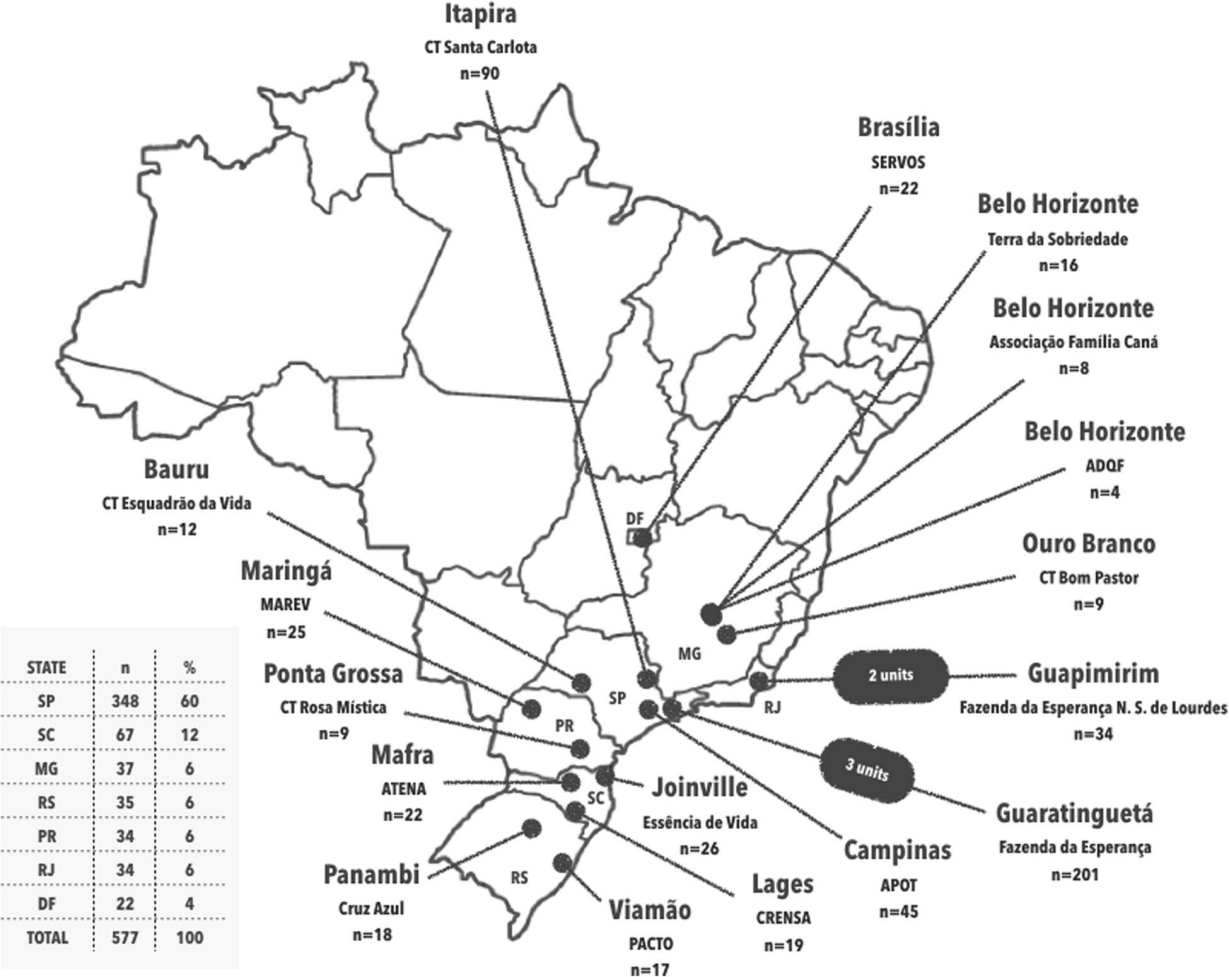
Brazilian political map showing were the 20 therapeutic communities involved in the current study are located. The Map of Brazil was obtained through the public domain website: http: //www.dominiopublico.gov.br/pesquisa/PesquisaObraForm.jsp, and the Apple Keynote software was used for the figure art

This is a cross-sectional study which included TCs (*n* = 20) affiliated with the Brazilian Federation of Therapeutic Communities (FEBRACT), which aims to train the Brazilian TCs according to the recommendations of the World Federation of Therapeutic Communities (WFTC). All TCs were registered with the Brazilian National Secretariat for Drug Policy (SENAD) and compliant with Brazilian Health Regulatory Agency (ANVISA) standards [34], this guaranteed the inclusion of study participants from TCs with similar standards of treatment. The following inclusion criteria were chosen to select the institutions: (1) at least 10 years of existence and (2) having a qualified and regularly trained health team. Only voluntary admissions were allowed in the included TCs; all patients who needed intensive medical/psychiatric care were referred to another health service. The geographic distribution of TCs are shown in Fig. 1. The study was restricted to the south region of Brazil due to logistic issues (e.g. internet access and professional staff training). The period of recruitment was 05 September 2012 to 05 September 2013.

### Participants

During the 12-month period of the study, a total of 1341 individuals presented at one of 20 TCs with a substance use disorder. Of those, 719 crack-cocaine users were voluntarily admitted with the diagnosis of crack-cocaine use disorders (F14.2) in accordance with the International Classification of Diseases 10th (ICD-10) [35]. Among those, twenty-five patients (3.5%) refused to participate, while another 117 (16.3%) signed the consent form but interrupted treatment almost immediately, leaving at the end 577 (80.2%) patients eligible to participate in the study.

### Instruments

A detailed structured interview was developed to be used in this survey (in supplementary file). It was designed to obtain baseline data on factors associated with crack-cocaine early initiation and other related behaviors, as well as information about parental monitoring in childhood, peer pressure and deviant behaviors. To organize the information collected and to formulate questions in tune with the international literature, we selected three structured questionnaires widely used in other studies. Sociodemographic data was selected from the Maudsley Addiction Profile (MAP) questionnaire [36], a brief research instrument developed in the United Kingdom, assessing four domains: substance use, health risk behavior, physical and psychological health, and personal/social functioning.

The questions on “parent relationship” (Table 1), “parent monitoring” (Table 2), and “perceptions related to easiness in accessibility and permissiveness of drug consumption” (Table 3) were extracted and translated from the Kenneth Kendler’s Life History Calendar Interview (from Stress and Coping Project, Medical College of Virginia) [17, 20]. Finally, questions on “deviant behaviors” (Table 4), were extracted and adapted from either the Drug Abuse Treatment Outcomes Study (DATOS) questionnaire [37] or from the referred Kendler’s interview.

**Table 1:** Family relationships (*n* = 577)

Characteristic	N (%)	Average age of first crack use	SD (CI 95%)	***P***-value^**a**^	Adjusted ***p***-value^**b**^
Lived continuously with his/her mother until 17 years old (*n* = 541)					
Yes	389 (71.9)	21.9	6.86 (19.5–21.7)	0.061	-^c^
No	152 (28.1)	20.6	6.98 (21.2–22.6)		
Until the age of 17, did you experience serious relationships problems with your mother for at least one month? (*n* = 387)					
Yes	143 (36.9)	20.5	6.36 (19.4–21.5)	**0.002**	0.466
No	244 (63.1)	22.7	7.22 (21.8–23.6)		
Lived continuously with his/her father until 17 years old (*n* = 544)					
Yes	292 (53.6)	22.5	7.06 (21.7–23.3)	**0.001**	-^c^
No	252 (46.4)	20.3	6.38 (19.6–21.1)		
Until the age of 17, did you experience serious relationships problems with your father for at least one month? (*n* = 291)					
Yes	123 (42.1)	20.9	5.73 (19.9–21.7)	**0.001**	-^c^
No	168 (57.9)	23.6	7.73 (22.4–24.8)		
From 8 to 18, how often did your parents or stepparents yell at each other? (*n* = 415)					
Never / rarely / sometimes	298 (71.8)	22.2	6.91 (20.8–23.6)	0.691	0.320
Often	117 (28.2)	21.8	6.54 (20.6–23.0)		
From 8 to 18, how often did your parents or stepparents physically assaulted each other? (*n* = 417)					
Never / rarely / sometimes	272 (89.2)	22.1	6.97 (21.3–23.0)	0.824	0.570
Often	45 (10.8)	21.9	6.89 (19.8–24.0)		
Have you witnessed fights with physical aggression between parents or caregivers? (*n* = 539)					
Yes	236 (43.8)	20.8	6.32 (20.0–21.6)	**0.038**	-^c^
No	303 (56.2)	22.1	7.39 (21.2–22.9)		
Did your father have trouble related to alcohol consumption? (*n* = 555)					
Yes	297 (53.5)	20.6	6.47 (19.9–21.3)	**0.001**	0.210
No	258 (46.5)	22.6	7.48 (21.7–23.5)		
Did your mother have trouble related to alcohol consumption? (*n* = 546)					
Yes	82 (15)	21.1	7.22 (19.5–22.7)	0.491	0.620
No	464 (85)	21.7	7.03 (21.1–22.4)		
During childhood or adolescence, have you ever felt yourself extremely mistreated – without food, shelter, medical care and deprived of your basic physical and emotional needs? (*n* = 570)					
Yes	106 (18.5)	20.1	6.52 (18.9–21.4)	**0.016**	0.309
No	464 (81.5)	21.9	7.08 (21.3–22.6)		
During childhood or adolescence, have you ever run away from home during the night? (*n* = 569)					
Yes	213 (37.4)	20.4	6.87 (19.5–21.3)	**0.001**	0.185
No	464 (62.6)	22.3	7.00 (21.6–23.0)		

SD = standard deviation. CI = confidence Interval. Significant results (*p* < 0.05) are bolded

^a^*t-*test for independent variables

^b^ANOVA, analyses adjusted by Bonferroni test

^c^Variables that are no longer binary after ANOVA

**Table 2:** Parent monitoring through late childhood and adolescence

Did your parents / caregivers really know:	8–11 years (***n*** = 542)				12–14 years (***n*** = 526)				15-17 years (***n*** = 483)			
	No | A little	Very well	*P*-value^a^	Adjusted p-value^b^	No | A little	Very well	*P*-value^a^	Adjusted p-value^b^	No | A little	Very well	*P*-value^a^	Adjusted p-value^b^
Who your friends were?												
N (%)	282 (52)	260 (48)			355 (67.4)	171 (32.6)			379 (79.5)	98 (20.5)		
Average age (SD)	20.9 (6.42)	22.5 (7.44)	**0.008**	0.392	20.6 (6.12)	23.6 (7.76)	**< 0.001**	0.097	21.7 (7.13)	21.0 (6.18)	0.371	0.725
CI 95%	20.1–21.6	21.6–23.4			20.0–21.2	22.4–24.7			21.0–22.4	19.7–22.2		
How you spent your money?^1^												
N (%)	157 (43.2)	207 (56.8)	0.113		309 (65.5)	163 (34.5)			394 (83.4)	79 (16.6)		0.423
Average age (SD)	22.4 (7.78)	21.2 (6.39)		0.768	20.3 (6.12)	23.1 (7.16)	**< 0.001**	0.061	21.7 (7.22)	21.1 (6.13)	0.444	
CI 95%	21.2–23.7	20.4–22.1			19.6–21.0	22.0–24.2			21.0–22.4	19.7–22.4		
Where you used to go in your spare time?												
N (%)	250 (46.2)	291 (53.8)	**0.001**		370 (70)	158 (30)			402 (83.4)	80 (16.6)		
Average age (SD)	20.6 (6.38)	22.6 (7.39)		0.503	20.5 (6.12)	24.2 (7.97)	**< 0.001**	**0.023**	21.9 (7.16)	20.2 (6.08)	0.059	0.856
CI 95%	19.8–21.3	21.8–23.5			19.9–21.2	23.0–25.5			21.2–22.6	18.9–21.6		

**(1)** 178 participants between 8 and 11 years of age, 53, between 12 and 14 years of age and 8 between 15 and 18 years of age reported no access to money at all by that time

*SD* Standard deviation. *CI* Confidence Interval. Significant results (*p* < 0.05) are bolded

^a^*t-*test for independent variables

^b^ANOVA, analyses adjusted by Bonferroni test

**Table 3:** Perceptions related to easiness in accessibility and permissiveness for consumption

Between 15 and 17, how easily did you get:	**Very easy | Easy**	**Hard | Very hard**	***P*-value**^**a**^	**Adjusted** ***p*-value**^**b**^
Cigarettes (tobacco) (*n* = 564)	557 (98.7)	7 (1.3)		
Average age (SD, CI 95%)	21.5 (6.89, 20.9–22.0)	24.0 (11.61, 11.8–36.1)	0.382	0.128
Alcohol drinks (*n* = 564)	547 (97)	17 (3)		
Average age (SD, CI 95%)	21.4 (6.82, 20.8–22.0)	25.5 (9.80, 20.3–30.7)	**0.020**	0.441
Marijuana (*n* = 561)	501 (89.3)	60 (10.7)		
Average age (SD, CI 95%)	20.9 (6.62, 20.3–21.5)	26.8 (7.52, 24.8–28.7)	**< 0.001**	**0.001**
Cocaine (“snorted”) (*n* = 560)	388 (69.3)	172 (30.7)		
Average age (SD, CI 95%)	20.1 (6.24, 19.4–20.7)	24.9 (7.36, 23.8–26.0)	**< 0.001**	0.816
Crack cocaine (*n* = 559)	221 (39.5)	338 (60.5)		
Average age (SD, CI 95%)	17.1 (4.21, 16.5–17.6)	24.5 (6.87, 23.7–25.2)	**< 0.001**	**< 0.001**
During childhood and adolescence, did your parents or caregivers find normal an adolescent:	**Yes | Partially**	**No**	***P*-value**^**a**^	**Adjusted** ***p*-value**^**b**^
To smoke cigarettes (tobacco)? (*n* = 519)	131 (25.2)	388 (74.8)		
Average age (SD, CI 95%)	20.9 (7.17, 19.7–22.1)	22.0 (6.81, 21.3–22.7)	0.109	0.192
To drink alcoholic beverages? (*n* = 520)	154 (29.5)	366 (70.5)		
Average age (SD, CI 95%)	21.4 (6.72, 20.3–22.4)	21.9 (6.96, 22.1–22.6)	0.468	0.866
To get drunk? (*n* = 520)	70 (13.4)	450 (86.6)		
Average age (SD, CI 95%)	21.2 (6.95, 19.6–22.9)	21.8 (6.90, 21.2–22.5)	**0.047**	0.949
To smoke marijuana? (*n* = 521)	18 (3.4)	503 (96.6)		
Average age (SD, CI 95%)	20.7 (7.34, 17.0–24.3)	21.8 (6.89, 21.2–22.4)	0.498	0.783
During childhood / adolescence, how often did your parents / caregivers:	**Almost everyday | Weekly**	**Monthly | Never**	***P*-value**^**a**^	**Adjusted** ***p*-value**^**b**^
Smoke cigarettes (tobacco) (*n* = 519)	380 (73.2)	139 (26.8)		
Average age (SD, CI 95%)	22.0 (7.17, 21.3–22.8)	20.8 (6.05, 19.8–21.9)	0.081	0.063
Drink alcoholic beverages (*n* = 521)	299 (57.3)	222 (42.7)		
Average age (SD, CI 95%)	21.7 (6.64, 20.9–22.4)	21.8 (7.24, 20.9–22.8)	0.799	0.366
Get drunk (*n* = 519)	187 (36.1)	332 (63.9)		
Average age (SD, CI 95%)	20.9 (6.19, 20.0–21.8)	22.2 (7.22, 21.4–23.0)	**0.047**	**0.034**
Smoke marijuana (*n* = 516)	48 (9.2)	468 (90.8)		
Average age (SD, CI 95%)	20.5 (6.52, 18.6–22.4)	21.9 (6.94, 21.2–22.5)	0.198	0.337
Snorted cocaine (*n* = 515)	28 (5.4)	487 (94.6)		
Average age (SD, CI 95%)	19.8 (4.99, 17.8–21.7)	21.9 (7.01, 21.2–22.5)	0.122	0.471
Between 15 and 17, how many friends of yours used to:	**Nobody | A few | Some**	**Majority | Everybody**	***P*-value**^**a**^	**Adjusted** ***p*-value**^**b**^
Smoke cigarettes (tobacco) (*n* = 557)	216 (38.7)	341 (61.3)	**0.001**	0.019
Average age (SD, CI 95%)	22.6 (6.97, 21.7–23.6)	20.7 (6.77, 20.0–21.5)		
Drink alcohol (*n* = 557)	201 (37)	356 (63)	**< 0.001**	0.027
Average age (SD, CI 95%)	23.3 (7.57, 22.3–24.4)	20.4 (6.27, 19.8–21.1)		
Get drunk (*n* = 557)	264 (47)	295 (53)	**< 0.001**	0.837
Average age (SD, CI 95%)	22.9 (7.27, 22.0–23.8)	20.2 (6.29, 19.4–20.9)		
Have problems related to alcohol use (*n* = 553)	324 (58.5)	229 (41.5)	**< 0.001**	0.928
Average age (SD, CI 95%)	22.5 (7.32, 21.7–23.4)	20.0 (5.99, 19.3–20.8)		
Smoke marijuana (*n* = 556)	283 (50.9)	273 (49.1)	**< 0.001**	0.109
Average age (SD, CI 95%)	23.0 (7.07, 22.2–23.8)	19.9 (6.36, 19.1–20.6)		
Smoke crack cocaine (*n* = 555)	498 (89.7)	57 (10.3)	**< 0.001**	**< 0.001**
Average age (SD, CI 95%)	22.2 (6.84, 21.6–22.8)	15.4 (3.89, 14.4–16.4)		

*SD* Standard deviation. *CI* Confidence Interval. Significant results (*p* < 0.05) are bolded

^a^*t-*test for independent variables

^b^ANOVA, analyses adjusted by Bonferroni test

**Table 4:** Deviant behavior

Characteristic	N (%)	Average age of first crack use	SD (CI 95%)	***P***-value^**a**^	Adjusted ***p***-value^**b**^
Until the age of 15, have you ever threatened someone with a gun? (*n* = 567)				**< 0.001**	**0.001**
Yes	172 (30.3)	19.4	5.8 (18.5–20.2)		
No	395 (69.7)	22.5	7.3 (21.8–23.3)		
Until the age of 15, have you ever hurt an animal on purpose – out of hunting (*n* = 566)				0.254	0.886
Yes	198 (35)	21.1	6.5 (20.2–22.0)		
No	368 (65)	21.8	7.3 (21.1–22.6)		
Until the age of 15, did you use to tell many lies? (*n* = 566)				**0.008**	0.484
Yes	406 (71.7)	21.1	6.8 (20.4–21.8)		
No	160 (28.3)	22.8	7.5 (21.6–24.0)		
Until the age of 15, you used to steal things from stores, children or your parents (*n* = 566)				**0.001**	0.011
Yes	283 (50)	20.5	6.2 (19.7–21.2)		
No	283 (50)	22.7	7.6 (21.8–23.6)		
Until the age of 15, have you ever assaulted a child to the point of sending him to the hospital? (*n* = 565)		20.9		0.206	0.442
Yes	141 (25)	21.8	7.2 (19.7–22.1)		
No	424 (75)		7.0 (21.1–22.5)		
Until the age of 18, have you been in a youth detention center? (*n* = 571)				**0.001**	0.050
Yes	95 (16.6)	19.0	7.2 (21.5–22.7)		
No	476 (83.4)	22.1	6.0 (17.8–20.3)		
Until the age of 18, have you been in a youth detention center for at least 3 months? (*n* = 570)				**0.005**	0.392
Yes	32 (5.6)	18.2	4.2 (16.7–19.7)		
No	538 (94.4)	21.7	7.1 (21.1–22.3)		

*SD* Standard deviation. *CI* Confidence Interval. Significant results (*p* < 0.05) are bolded

^a^*t-*test for independent variables

^b^ANOVA, analyses adjusted by Bonferroni test

Participants were asked to recall the above issues considering three age periods: 8 to 11 (middle childhood), 12 to 14 (early adolescence) and 15 to 17 (middle adolescence), as previously used by Kendler’s study group [25]. *Middle childhood* is a period marked by progressive independence from parents, greater concern with the future and stronger and more complex friendships and peer relationships. It is a critical period for the development of trust and intimacy outside the family circle - among friends in the neighborhood, at school and during sports practice. A rapid development of cognitive skills allows children to develop a sense of responsibility and learn more elaborate ways to communicate their ideas and feelings. *Early adolescence* is characterized by rapid growth and body changes, including genital development, which inspires curiosity and increasing feelings for privacy. Early adolescents thinking works essentially in a concrete way - “right-or-wrong thinking” - and it is structured partly from the idea of being always judged by their peers. During *middle adolescence* the physical changes may be nearly completed. The interest for romantic and sexual relationships and the issues considering sexual identity progressively come to occupy the forefront of the teenagers’ lives. More elaborated forms of argumentation and more independent postures of life are usual at this stage. Adolescents between 15 and 17 years spend less time with family and more time with friends - peer pressure may peak at this stage. Abstract thinking, complex decision making, impulse control and capacity for otherness start to develop during this period [38, 39]. There are consistent research pointing that parental monitoring have different effects on child and adolescent behaviors, considering these age groups [40–46].

### Procedures

This structured interview had been extensively pilot tested beforehand. The pilot tests were carried out 1 month prior to data-collection, first among the researchers and then among the interviewers. Finally, as part of the pilot test phase, the interviewers conducted the structured interview with at least one recently admitted patient. All interviewers (*n* = 41) were health professionals - psychologist, social worker or nurse. The interviewers were trained locally by at least one member of the research group (*n* = 6). Remote assistance was offered for the interviewers to clarify any questions prior to launch of data-collection.

Interviews were conducted within the first 15 days following treatment admission. The interview day was decided based on an assessment of the participant’s condition, such as clinical status, abstinence syndrome, treatment adherence, willingness to be interviewed that time/day, among others. Interviews were conducted in a private room, in front of a computer, side by side with the patient, during two consecutive days and each of them lasting about 1 h and a half.

The interview and the adopted scales were incorporated into an electronic research data platform (Sphinx iQ2®) allowing real-time data entry and storage in an online database. The structured interview followed a pre-defined sequence and comprised multiple choice questions. The online platform was programmed to prevent invalid or unfilled responses, this guaranteed the standardization of the interview and avoided missing data. In order to monitor the quality of data collection, a member of the study team was designated to each study site and was responsible for verifying each new entry, as well as, for visiting the TC at least twice during the study period.

### Statistical analyses

To test differences between characteristics according to crack-cocaine early use, we used Student *t*-test for comparisons between groups. The *t*-test is implemented when the population variance, σ^2^, is generally unknown, and in this case, the sample variance, *s*^*2*^, is used and the *p*-value of the test will allow us to assess whether the average age difference for the beginning of crack-cocaine is significant at the 0.05 level. For each table, each categorical variable’s *t*-test was used to compare the average age at onset of crack-cocaine use by respondents who answered “yes” or “no” to each question. Analysis of variance (ANOVA) were used to compare the results of the variables provided in each table. Due to the multiple comparisons, Bonferroni tests were performed for statistical adjustment in regard to all variables. Subsequently, to test the independent effect of the risk and protective factors associated with early crack-cocaine initiation, a multiple linear regression model was performed only with variables that remained significant after Bonferroni’s adjustment. All analyses were conducted using the STATA, version 13.1.

## Results

Sociodemographic data of this study have been published in detail elsewhere [47]. Study participants were mainly male (*n* = 517, 89.6%), living alone (single, divorced or widow) (*n* = 448, 78.4%), with and average age of 30.8 years (SD = 7.7). Three-quarters (73.9%) were 34 years old or younger. They had a medium of 11.8 school years (SD = 4.4). Aspects related to religiosity have also already been published [48]. Average age of first use of crack-cocaine was 21.5 years (SD = 7.0).

### Family relationships

#### Quality of the relationships, neglect, maltreatment and violence

None of the variables in this subgroup reached significance after Bonferroni’s corrections – some of them reached only a nominally significant difference, considering the average age of onset of crack-cocaine use between groups. A significant earlier onset of crack-cocaine use was observed when the father had problems with alcohol use (*p* = 0.001; Bonferroni corrected *p* = 0.210) or when the participant had a constant relationship conflict with the mother (*p* = 0.002; Bonferroni corrected *p* = 0.466) or the father (*p* = 0.001). The presence of an extremely severe maltreatment episode during childhood or adolescence (*p* = 0.016; Bonferroni corrected *p* = 0.309) or escaping home at night during the same period (*p* = 0.001; Bonferroni corrected *p* = 0.185) were related to early use of crack-cocaine. In addition, having witnessed physical aggression among the parents showed an association (*p* = 0.038).

### Parent or caregiver monitoring

A similar scenario occurred with the variables on parental monitoring after Bonferroni’s adjustments (Table 4). Only the variable related to monitoring of spare time in the 12 to 14 year old period remained significant as a protective factor for later age of onset of crack-cocaine use (*p* = < 0.001; Bonferroni corrected *p* = 0.023). The other variables showed differences only in test *t-*Student analyzes. The study patients whose parents knew their friends (*p* = 0.008; Bonferroni corrected *p* = 0.392) and knew how they spent their free time when they were 8–11 years old (*p* = 0.001; Bonferroni corrected *p* = 0.503) started crack-cocaine consumption later. These same actions of parental monitoring added to the control of respondents’ expenses in early adolescence (12–14 years), remained associated as protective factors for the age of onset of crack-cocaine consumption (*p* < 0.001, for all variables; Bonferroni corrected *p* = 0.097; *p* = 0.023 and *p* = 0.061, respectively). However, parental monitoring was no longer a factor related to early age of onset from 15 years old onwards.

### Accessibility and drug use permissiveness by parents

#### Perception related to easiness in accessibility for consumption

During adolescence, the perception related to easy accessibility of marijuana (*p* < 0.001; Bonferroni corrected *p* = 0.001) and crack-cocaine (*p* < 0.001, for both analyses) remained statistically significant after Bonferroni’s adjustments (Table 2).

#### Permissiveness of alcohol, tobacco and drug use at home

The results showed that parents finding “normal” for their children, as well as for themselves, to use alcohol, tobacco and marijuana anywhere was not a predictive factor for early initiation of crack-cocaine use (Table 2). An association was observed between those reporting having parents who got drunk daily/weekly and using crack-cocaine early (*p* = 0.047; Bonferroni corrected *p* = 0.034).

#### Peer factors

Having the “majority/all” of friends using crack-cocaine was associated with early crack-cocaine use, and this association remained significant after Bonferroni’s correction (*p* < 0.001). For the other types of drugs investigated - tobacco (*p* = 0.001; Bonferroni corrected *p* = 0.019), alcohol (*p* < 0.001; Bonferroni corrected *p* = 0.027), marijuana (*p* < 0.001; Bonferroni corrected *p* = 0.109), as well as for patterns of consumption - to get drunk, problematic alcohol use (*p* < 0.001, for both cases; Bonferroni corrected *p* = 0.837 and *p* = 0.928, respectively) - there were significant differences only in the results of the Student *t* test analyses (Table 2).

### Deviance behavior and contact with youth justice system (before 15 years old)

Reporting to have threaten someone with a gun before the age of 15 showed a significant interaction effect with the age of onset of crack-cocaine use (*p* < 0.001; Bonferroni corrected *p* = 0.001). Telling many lies (*p* = 0.008; Bonferroni corrected *p* = 0.484) and steal stores or their parents (*p* = 0.001; Bonferroni corrected *p* = 0.011), as well as, having been in a detention center (*p* = 0.001; Bonferroni corrected *p* = 0.050), regardless of the amount of time (*p* = 0.005; Bonferroni corrected *p* = 0.392) were significant only for the results of the Student *t* test analyses.

### Risk and protective factors associated with initial crack-cocaine use

In the multivariate model (Table 5) variables were selected using the statistical criteria explained above (i.e., those remaining significant after Bonferroni’s adjustment): parental monitoring of places where the patients spent their free time during ages of 12–14 years, perceptions related to ease access to get marijuana and crack-cocaine during adolescence, frequency of drunkenness of parents or caregivers, having peer crack-cocaine users in adolescence, and deviant behavior (e.g., threatening someone with a gun) before the age of 15. This second model showed that parental monitoring delayed the start of crack-cocaine use by approximately 1.25 years (*p* = 0.043, 95% CI = 0.41, 2.50); ease-of-access to marijuana decreased the age of onset by an average of 2 years (*p* = 0.028, 95% CI = − 3.81, − 0.22), while access to crack-cocaine by about 6 years (*p* < 0.001, 95% CI = − 7.40, − 4.90). In addition, the use of a gun before the age of 15, decreased the age of starting crack-cocaine by approximately 1.3 years (*p* = 0.028, 95% CI = − 2.57, − 0.14), while the remaining variables were not found to be significant.

**Table 5:** Risk and protective factors associated with initiation of crack-cocaine use (*n* = 479)

	***P***-value^**a**^	95% CI
Did your parents/caregivers really know:		
Where you used to go in your spare time? (12–14 years) (*n* = 526)	**0.043**	0.04, 2.50
Between 15 and 17 years of age, how easily could you access:		
Marijuana (*n* = 561)	**0.028**	−3.81, −0.22
Between 15 and 17 years of age, how easily could you access:		
Crack-cocaine (*n* = 559)	**< 0.001**	−7.40, −4.90
During childhood/adolescence, how often did your parents/caregivers:		
Get drunk (*n* = 519)	0.602	−0.82, 1.42
Between 15 and 17 years of age, how many friends of yours used to:		
Smoke crack-cocaine (*n* = 555)	0.267	−3.02, 0.83
Before the age of 15, have you ever threatened someone with a gun? (*n* = 567)	**0.028**	−2.57, −0.14

*CI* Confidence Interval. Significant results (*p* < 0.05) are bolded

^a^Multiple linear regression model

## Discussion

This is the first multicenter study that investigated several risk and protective factors associated with the initiation of crack-cocaine use among individuals seeking treatment. Our results add new evidence on predictor factors for the early use of this substance. We found that involvement before the age of 15 in illegal acts - using a firearm to threaten someone - and the perception of easy access to illicit drugs during adolescence - marijuana and crack - were associated risk factors for the early initiation of the use of crack-cocaine, while the monitoring of free time by parents in the early years of adolescence was a protective factor and delayed the experimentation with the drug.

Although studies on the influence of the quality of family relationships at the initiation of illicit drug use are relatively scarce compared with other studies that evaluate these same influences for the initiation of licit drug use [49], it is believed that a supportive family environment with a strong bond with family members and a low level of family conflict can predict a lower risk of initiating drug use in adolescence [26, 49]. In this study, relationship problems with both parents, including violence between them, paternal absence and problematic alcohol consumption by the father were factors associated with the early onset of crack use; however, they did not have an independent effect when other family variables were analyzed together. In line with these findings, some studies have found that proximity to parents was associated with a significantly lower risk of starting to use illicit drugs; but they were also not predictors for starting illicit drug consumption when the effect of other variables was controlled [50, 51].

In the present study, only half of the patients felt adequately monitored during late childhood (8–11 years). This perception fell to less than a third - except for the “night walks” (41.2%) - between 12 and 14 years, and 20 to 26% during the last years of adolescence. Similarly, a cohort study followed a group of students (*n* = 808) for 11 years, interviewing them on seven occasions until they were 21 years old and noticed that parental monitoring was related to the low incidence of initiation to drug use only until the age of 15, when it ceased to function as an independent variable, when peer predictors were added [49, 52].

However, the absence of parental monitoring lost its significant effect when other variables were considered. This was not expected given the vast literature correlating parental monitoring as a substantial predictive effect on the age of initiation of licit and illicit substances [17, 18, 21, 30, 44–46, 49, 53, 54]. However, these results are consistent with other evidence. Previous studies have demonstrated associations between temperament, conduct problems, and substance use and abuse [44, 55–60]. In addition, longitudinal studies have observed that the low effect of parental monitoring in some samples of drug users may be linked to other specific individual issues such as genetic factors [52, 53, 61, 62] and gender [49, 54].

On the other hand, this study showed that monitoring of free time by parents during early adolescence (12–14 years) acted as a strong protective factor for delaying the initiation of crack-cocaine use. This finding is in line with recent research that seeks to define parental monitoring not only as a measure of how well informed parents are about their children or adolescents, but how much they actually supervise the behavior of their children [54, 63] and if parents know the places and the time spent with friends, after school activities.

Among all the variables in Table 2 - perceptions related to easiness in accessibility and permissiveness for consumption during adolescence - easy access to marijuana and crack, having parents who get drunk frequently and having friends who are crack-cocaine users were risk predictors for the early initiation of crack-cocaine use. Peer pressure is certainly one of the most striking influences, especially when it is appears predominantly in the social repertoire of the individual. Its magnitude is substantial from late adolescence (15 years), weakening after adulthood [17, 33, 49]. In the present study, those who reported having crack-cocaine friends in their teens started using crack-cocaine earlier. Kiesner and colleagues [54] investigated that although the evidence that peers play a central role in adolescent substance use, other important factors require further study, especially when adolescents and their friends engage in substance use together.

According to the National Epidemiologic Survey on Alcohol and Related Conditions (NESARC), the facilitated access to the different substances and peer pressure are important risk factors for the initiation of the use of drugs [33]. In our analyzes, access to crack-cocaine decreased the age of onset of use of the same substance by approximately 6 years, while access to marijuana by an average of 2 years. Many studies have already evaluated the use of marijuana as a predictor for the use and dependence of other illicit drugs [4, 49, 64]. Another survey showed that among young people with a cocaine exposure opportunity, those who had used marijuana were an estimated 15 times more likely to use cocaine than those with no history of marijuana [65].

Several studies support that manifesting deviant behavior before the age of 15 predicts future substances [40, 42, 44, 66]. The present study found that the use of firearms was a predictor for the early use of crack-cocaine. A complex association of early involvement with crime, peer pressure and access to illicit drugs during adolescence, remained strongly associated with early initiation of crack-cocaine use. These factors illustrate the intertwined relationship between deviant behaviors and increased risk for early initiation of psychoactive substances [67, 68]. A study with street boys in Canada pointed out that episodes of “early serious delinquency” (ESD) – at aged 12 or younger – such as using a weapon, breaking into a building or having stolen an expensive object was related to early drug initiation [14].

Similarly, studies interested in observing predictive factors of the first episode of drug use have noted that some deviant behaviors can be inhibited in the presence of parental monitoring, this same effect was observed in antisocial behaviors related peer pressure [17, 33, 41]. In light of this, considerations could be given to promote the strengthening of the parent-child bond, as well as stimulate parental monitoring as a way to counteract the effects of peers on early crack-cocaine initiation.

### Strengths and limitations

This study has some potential limitations. It is a cross-sectional study which preclude us to establishment of causality from the analyzed variables and age at crack-cocaine initiation. Despite being a multicenter study from different Brazilian regions, participants were selected from a specific setting (treatment), and from a single treatment type (Therapeutic Communities), which limits external validity. Each interviewer had the opportunity to evaluate one patient at a time, which had implications for ‘inter-rater’ analyses based on statistical techniques. Nevertheless, criteria were established to ensure agreement between them, such as having a similar level of qualifications, and having been trained by one member of the research group. The interviews were based on retrospective data recalled by the patients, so the data collected is subjected to recall bias. However, self-reported drug use, collected with assured confidentiality, is considered a reliable and valid method [69–71], and the research team took specific efforts to help the interviewees to recall the required information. Finally, in addition to the variables investigated, other important and well-established factors in the international literature have not been assessed in depth, such as the presence of genetic factors and psychiatric disorders. Despite inherent limitations with longitudinal studies, they can nevertheless elucidate the causality of these variables and the outcome of crack-cocaine use.

## Conclusions

The abuse and dependence on crack-cocaine represents a challenge for our society, bringing countless losses at the individual and social levels. There is growing interest in research that seeks to find predictive risk factors for initiation of use as an effective way to prevent crack-cocaine addiction. The current study showed that accessibility factors to illicit drugs and involvement in deviant behaviors should be important factors for drug preventive efforts.

## Supplementary Information

**Additional file 1.**

## Data Availability

The datasets generated and/or analysed during the current study are not publicly available due to the confidentiality of personal information, but are available from the corresponding author on reasonable request.

## Abbreviations

ANOVA: Analysis of variance
ANVISA: Agency of health surveillance
BNADS: II Brazilian national alcohol and drugs survey
DATOS: Drug abuse treatment outcomes study
ESD: Early serious delinquency
FEBRACT: Brazilian federation of therapeutic communities
HIV: Human immunodeficiency viruses
ICD-10: International classification of diseases 10th
MAP: Maudsley addiction profile
NESARC: National epidemiologic survey on alcohol and related conditions
SAMHSA: Substance abuse and mental health services administration
SENAD: National secretariat for drug policy
TC: Therapeutic communities
WFTC: World federation of therapeutic communities
