# Supplementary material for: Factors associated with crack-cocaine early initiation: a Brazilian multicenter study
Source: BMC Public Health. 2021 Apr 23;21:781. doi: 10.1186/s12889-021-10769-x (PMC8063477; doi:10.1186/s12889-021-10769-x)
Supplement: Supplementary file 1 — Additional file 1. [file 12889_2021_10769_MOESM1_ESM.pdf]

SOCIODEMOGRAPHY

Age	Numeral
Gender	Male l Female
Race	White l No-white
Type of housing	House (own house or rented house) l Hotel, Pension l Medical institution l Prision, Children's shelter l Assited living l Hostel l Homeless - Street situation
Who did you live with when you were hospitalized?	Alone l With some family member l With friends l Closed institution l Without fixed residence
Provider	Himself/Herself l Relatives l Friends l Institutions

PARENTS AND CAREGIVERS

Where did you go after maternity?	With mother/relative l Institution l Adopted l Not know
Did you live with your mother until you were 17 years old?	Yes l No
Have you had serious relationship problems with your mother for at least a month until you were seventeen years old?	Yes l No
Did you have serious relationship problems with your mother until you were seventeen years old?	Yes l No
Why did not you live with your mother?	She left home l Divorce l She handed me to another relative l Death/disease l Adoption/shelter l I left home l I was arrest l Others
Patients who did not live with their mothers: Did you have someone who cared like a mother?	Yes l No
Did you have a stepfather?	Yes l No
Have you had serious problems with your stepfather for at least a month until you were 17 years old?	Yes l No

Have you had serious problems with your stepfather until you were 17 years old?	Yes l No
What was the reason you did not live with your stepfather any more?	I stayted with him l I left home l My stepfather left home
Reason for leaving the stepfather	Separation/Divorce l By chemical dependence of the stepfather l By conflicts and fights l Death l Others
Did you live with your father until you were 17 years old?	Yes l No
Have you had serious problems with your father for at least a month until you were 17 years old?	Yes l No
Have you had serious problems with your stepfather until you were 17 years old?	Yes l No
What was the reason you did not live with your father any more?	Divorce/Separation – includes: when the mother left home l Shelter/ adoption l Physical violence l exposure to domestic violence - physical aggression to mother or when the father had chemical dependence l Father left home l Financial conditions l I left home l I was left with family members l Father was arrested l Father was in street situation l I've never lived with my father l Death l Others
Patients who did not live with their fathers:<br>Did you have someone who cared like a father?	Yes l No
Did you have a stepmother?	Yes l No
Have you had serious problems with your stepmother for at least a month until you were 17 years old?	Yes l No
Have you had serious problems with your stepmother until you were 17 years old?	Yes l No

Did you live in a shelter from birth until your 17 years of age?	Yes l No
Adoption	Adoption l Biological l Not want to answer

RELATIONSHIP WITH PARENTS (BIO, ADOPTIVES, CAREGIVERS)

Have your parents separated for more than 6 months before your 18th birthday?	Yes I No
Did your mother die before your 18th birthday?	Yes I No
Did your father die before your 18th birthday?	Yes I No
Have you witnessed fights with physical aggression between your parents?	Yes I No
How often did you witness aggressions between your parents?	Only once I Rarely I Monthly I Weekly or more I Daily
Was there alcohol consumption by your mother or father or both at such times?	Never I Sometimes I Always

FAMILY HISTORY OF USE ALCOHOL AND OTHER DRUGS

Mother user	Yes I No
Mother – type of psychoative substance	Alcohol I Drugs I Both
Father	Yes I No
Father – type of psychoative substance	Alcohol I Drugs I Both

INTERPERSONAL RELATIONSHIPS IN THE LAST YEAR BEFORE HOSPITALIZATION

Last year did you usually communicate with your family and friends?	Not once I Few times I Always
Had more intimate problems?	Never I Few times I Often
How often did your family or friends help or encourage you to stop using drugs?	Never I Few times I Often

How often did your family or friends help you with employment, legal problems, etc.?	Never l Few times l Often
Did you exercise, have hobbies, groups of friends or go to a club?	Nothing l < weekly l At least once a week l Almost daily
Have people close to you using illegal drugs?	None l Few l Many
Were there people close to you who drank too much?	None l Few l Many
How many people close to you have been arrested?	None l Few l Many
How many friends started treatment last year?	None l Few l Many

LIFE SITUATIONS BEFORE 15 YEARS OF AGE

Have you ever felt extremely ill-treated, without food, without shelter, without medical care, without the basic physical and emotional needs?	Yes l No
Did you ever run away from home during the night?	Yes l No
Did you live with your parents after that?	Yes l No
Were you a shy child or shy teenager?	Yes l No
Did you get into fights you started yourself?	Yes l No
Have you ever threatened anyone with any kind of weapon?	Yes l No
Have you ever intentionally injured an animal?	Yes l No
Did you tell a lot of lies?	Yes l No
Did you steal things from stores, from other children, or from your parents?	Yes l No
Have you ever assaulted a child to the point of sending her to the hospital?	Yes l No

LIFE SITUATIONS BEFORE 15 YEARS OF AGE

Have you spent at least 3 months in a socio-educational center such as the Casa Foundation?	Yes l No

LIFE SITUATIONS BETWEEN 18 AND 30 YEARS OF AGE

Have you ever been arrested or arrested awaiting a lawsuit?	Yes l No

EDUCATION

Educacional Level	Never studied l Primary incomplete l Primary complete l Secondary incomplete l Secondary complete
Have you repeated your year in school?	Yes l No
How many times?	1 l 2 l 3 l 4 l 5 l >5
Did you have problems with teachers because of misbehavior?	Yes l No
Have you been suspended or expelled for misbehavior?	Yes l No
Have you already "killed class" (missing class because of irresponsibility)?	Yes l No
Do you have a technical course?	Yes l No
Did you complete college?	Yes l No

MARITAL STATUS

Marital status	Single, Divorced, widower l Married
Do you have kids?	Yes l No
Do you have adopted children?	Yes l No
Do you have stepchildren?	Yes l No

JOB

Do you have or have you ever had regular work for more than 6 months?	Yes I No
Were you working regularly up to 30 days before your hospitalization?	Yes I No
What is your current employment relationship?	Registered I Self-employed I Informal
Why was not he working?	Demission I Removed I Retired
Unemployment time	1 or less I 1-6 months I 7-12 I 12 – 24 I >24
Reasons for demission	For alcohol and drugs I Others
Reasons for removed	For alcohol ans drugs I Others
In the last week before admission, her main activity was?	Fully licit employment I Employment, without work I Looking for work I Taking care of the home for the family or for themselves I Studying I Incapacitated for work I Retired I Prisoner I Interned elsewhere I Traffic I Other illicit activities I Without work and without seeking employment I Not want to answer

HISTORY
The same questions were asked for different age groups:

BETWEEN 8-11 YEARS OLD
BETWEEN 12 – 14 YEARS OLD
BETWEEN 15 – 17 YEARS OLD

Did you live with your parents or caregivers for a year?	Yes I No
How often did you go through quarrels, disagreements or conflicts **with your mother**?	Almost daily I Few times a week I Weekly I Monthly I A few times in the year I Never
How often did you go through quarrels, disagreements or conflicts **with your father**?	Almost daily I Few times a week I Weekly I Monthly I Sometimes in the year I Never
Overall how was the relationship you had **with your mother**?	Very close I Some proximity I A bit close I Not too close I Nothing near

Overall how was the relationship you had **with your father**?	Very close I Some proximity I A bit close I Not too close I Nothing near

Did your parents really know their friends?	No, A little I Very well
Did your parents know how you spent your money?	No, They knew little I They knew well I I had not money
Did your parents know what you did in your free time?	No,They knew little I They knew a lot

How often did you participate in regular sports activities in or out of school?	Never I Rarely I Sometimes I Frequently
How often did you participate in extracurricular activities - courses, cultural activities?	Never I Rarely I Sometimes I Frequently
How often did you attend a religion?	Never I Less than 1 time per month I Few times in the month I Once a week I More than once a week I Once a month I Do not remember
How often did you participate in activities related to any religion?	Never I Rarely I Sometimes I Frequently
How was your performance at school?	High above average I Better than average I On average I Below average I Not want to answer

How many of your friends **smoked cigarettes**?	None, Few, Some I Most, All
How many of your friends **consumed alcohol**?	None, Few, Some I Most, All
How many of your friends **got drunk**?	None, Few, Some I Most, All
How many of his friends **stole, robbed, or destroyed the property of others**?	None I Few I Some I Most I All
How many of your friends **intentionally missed the class**?	None I Few I Some I Most I All
How many of your friends **"glued" to the tests** (copy the colleague's test)?	None I Few I Some I Most I All
How many of your friends **smoked marijuana**?	None I Few I Some I Most I All
How many of your friends **used other drugs**?	None I Few I Some I Most I All

How many of your friends **used crack**?	None, Few, Some I Most, All

How often did you **get into fights**?	Often I Sometimes I Rarely I Never
How often did you **tell lies**?	Often I Sometimes, Rarely,Never
How often have you **stolen things from a store or someone, including family members**?	Never I 1-2 times, 3-5 times, more than 6
How often have you **seriously injured or injured someone**?	Never I 1-2 times, 3-5 times, more than 6
How often did you **run away from home during the night**?	Never I 1-2 times, 3-5 times, more than 6
How often did you **deliberately start a fire knowing that you should not do it**?	Never I 1-2 times I 3-5 times I more than 6
How often have you **purposely destroyed someone's property?**	Never I 1-2 times I 3-5 times I more than 6
How often have you **physically injured animals - except hunting or fishing**?	Never I 1-2 times, 3-5 times, more than 6
How often did you **steal or cheat anyone**?	Never I 1-2 times, 3-5 times, more than 6

HOW EASILY COULD YOU GET THE FOLLOWING SUBSTANCES IF YOU WANTED TO CONSUME THEM, EVEN FOR THE FIRST TIME?

Cigarettes	Very easy, Easy I Difficult, Very difficult
Alcohol	Very easy, Easy I Difficult, Very difficult
Solvent	Very easy, Easy I Difficult, Very difficult
Cannabis	Very easy, Easy I Difficult, Very difficult
Cocaine	Very easy, Easy I Difficult, Very difficult
Crack	Very easy, Easy I Difficult, Very difficult
Amphetamines	Very easy, Easy I Difficult, Very difficult

PARENT’S ATTITUDE DURING CHILDHOOD-ADOLESCENCE

Have you lived with your parents or caregivers for at least 5 years between your 8-18 years of age?	Yesl No
Did your parents find it normal for a **teenager to smoke cigarettes**?	Yes, Partly l No
Did your parents think it normal for **a teenager to drink alcohol**?	Yes, Partly l No
Did your parents think it normal for **a teenager to get drunk**?	Yes, Partly l No
Did your parents think it normal for **a teenager to smoke marijuana**?	Yes, Partly l No
Did your parents consider it normal for **a teenager to use drugs like cocaine and LSD**?	Yes, Partly l No

How often did your parents and other adults who lived in your home **smoke cigarettes**?	Every day or almost, 1-2 times a week l A few times in the month, 1-2 times a year, Never
How often did your parents and other adults who lived in your home **consume alcohol**?	Every day or almost, 1-2 times a week l A few times in the month, 1-2 times a year, Never
How often did your parents and other adults who lived in your home **get drunk**?	Every day or almost, 1-2 times a week l A few times in the month, 1-2 times a year, Never
How often did your parents and other adults who lived in your home **smoke marijuana**?	Every day or almost, 1-2 times a week l A few times in the month, 1-2 times a year, Never
How often did your parents and other adults who lived in your home **use cocaine in powder form**?	Every day or almost, 1-2 times a week l A few times in the month, 1-2 times a year l Never
How often did your parents and other adults who lived in your home **smoke crack**?	Every day or almost, 1-2 times a week l A few times in the month, Never

Between your 8-18 years, have you lived with your parents/ stepparent married for at least 5 years?	Yes I No
How often did your parents **fight with each other**?	Never, Rarely, Sometimes I Frequently
How often did your parents **yell at each other**?	Never, Rarely, At times I Frequently
How often did your parents **beat each other**?	Never, Rarely, At times I Frequently

Did you have difficulty carrying out school activities and homework carefully?	Never I Rarely I At times I Frequently
Did you have difficulty sitting still?	Never I Rarely I At times I Frequently
Were you "daydreaming" or thinking about other things while you were at school or doing your homework?	Never I Rarely I At times I Frequently
Did you lose patience with your parents, other adults or teachers?	Never I Rarely I At other times I Frequently
Did you forget the things you should do?	Never I Rarely I At times I Frequently
Did you interrupt or intrude on the activities of others?	Never I Rarely I At other times I Frequently
Did you have difficulty performing your work when other things happened around you?	Never I Rarely I At times I Frequently
Did you refuse to do things that your parents or teachers asked you to do?	Never I Rarely I At times I Frequently
Did you change what you were doing without finishing what you started?	Never I Rarely I At times I Frequently
Did you have difficulty staying in line?	Never I Rarely I At times I Frequently
Did you lose things?	Never I Rarely I At times I Frequently

Did you get any recovery from school?	Yes I No
Have you repeated a year at school?	Yes I No

How many years did you repeat in school?	1 I 2 I 3 I 4 I 5 I 6 or more

The same questions were asked for different age groups:

BETWEEN 18 – 21 YEARS OLD
BETWEEN 22 – 25 YEARS OLD

How many of your friends **smoked cigarettes**?	None, Few, Some I Most , All
How many of your friends **consumed alcohol**?	None, Few, Some I Most, All
How many of your friends **got drunk**?	None, Few, Some I Most, All
How many of your friends had **alcohol-related problems- fights, hangover**?	None, Few I Some, Most, All
How many of his friends **stole, robbed, or destroyed the property of others**?	None I Few I Some I Most I All
How many of your friends had **problems with the law**?	None I Few I Some I Most I All
How many of your friends **smoked marijuana**?	None, Few, Some I Most, All
How many of your friends **used solvents**?	None I Few I Some I Most I All
How many of your friends **used other drugs**?	None I Few I Some I Most I All
How many of your friends **used crack**?	None, Few, Some I Most, All
How many of your friends **gave or sold drugs to other kids**?	None I Few I Some I Most I All

How easily could you get the following substances if you wanted to consume them, even for the first time?

Alcohol	Very easy, Easy I Difficult, Very difficult
Cannabis	Very easy, Easy I Difficult, Very difficult
Cocaine	Very easy, Easy I Difficult, Very difficult
Crack	Very easy, Easy I Difficult, Very difficult

Amphetamines	Very easy, Easy I Difficult, Very difficult

PERSONALITY

It pleases me and it excites me to go through new experiences and sensations, however much they cause a little fear	True I False
I would like to make a trip without any type of planning or prior choice of road map or date definition	True I False
I like to do things only when there is something exciting in them	True I False
I tend to change my interests all the time	True I False
Sometimes I like to do things that cause a little fear	True I False
I like the kind of life where people move all the time, with a lot of change and enthusiasm	True I False
Sometimes I like to do "crazy things" just to have fun	True I False
I like to go to the strange sides of the city with my own legs, even if I get lost	True I False
I like friends who are excitingly unpredictable	True I False

PSYCHOACTIVE SUBSTANCE

CRACK

Age of first consumption	Numeral
Age when started using weekly	Numeral
Age when started using daily	Numeral
How old were you when you smoked more crack?	Numeral

Did you use crack with marijuana?	Yes l No
At the stage where you most smoked, did you consume crack?	<monthly l biweekly l weekly l 2 times a week l Almost every day l Every day l More than once a day

In the phase where you smoked most, how long did your crack use last?	A few hours l All night long l More than one day l Several days
In the phase you smoked most, what was the maximum amount (rocks) you consumed in one go?	1-5 l 6-10 l 11-20 l 20-30 l >30
At the stage where you most smoked, did you use crack with someone else?	Always alone l Usually alone l Usually with someone l Always with someone

OTHERS PSYCHOACTIVE SUBSTANCE

INTRANASAL COCAINE ("SNIFFING")

Have you ever in your life you "sniffed" cocaine?	Yes l No
Age of first consumption	Numeral
Age when started using regularly	Numeral
Age when started using daily	Numeral
How old were you at the stage where you used most cocaine	Numeral
At the stage where you most sniffed, was your consumption?	<monthly l biweekly l Weekly l 2 times a week l Almost every day l Every day l More than once a day
How often do you think you sniffed cocaine?	1-2 l 3-5 l 6-10 l 11-49 l 50-99 l 100-199 l >200
In the phase where you smoked most, how long did your cocaine use last?	A few hours l All the night l More than one day l used in small amounts without stopping what I was doing
At the stage where you most smoked, did you use cocaine with someone else?	Always alone l Usually alone l Usually with someone l Always with someone

In the phase you smoked most, what was the maximum amount you consumed in one go?	<1 I 1-5 I 6-10 I >10
Since you started smoking crack, how was your cocaine use sniffed?	Disappeared I Decreased I Not changed I Increased

INJECTABLE COCAINE

Have you ever injected cocaine in your life?	Yes I No
Age of first consumption	Numeral
How old were you at the stage where you used most cocaine?	Numeral
At the stage where you most injected cocaine, was your consumption?	<monthly I Biweekly I Weekly I 2 times a week I Almost every day I Every day I More than once a day
When was the last time you injected cocaine?	<1 month I 1-5 months I 6-11 months I 12 months or more
Have you ever shared syringes?	Never I 1 time only I rarely I usually

TABACCO

Have you ever smoked?	Yes I No
Age of first consumption	Numeral
Age at which the first cigarette smoked	Numeral
How old were you when you started smoking regularly	Numeral
What was the highest amount of cigarettes you ever smoked?	< 10 I 10-19 I 20-40 I 41-60 I 61-80 I >80 I I Never smoked
Have you smoked regularly in the last month?	Yes I No I Never smoked
When did you smoke the most, did you wake up at night to smoke?	Yes I No I Never smoked
How often did this happen?	Weekly I 1-2 times a week I 3-5 times I 6-7 I more than once a night I Do not wake up I Never smoked

Ever tried to stop smoking seriously?	Yes I No I Never smoked
How often?	1-3 I 4-10 I >10 I Never tried I Never smoked
**APPLICATION OF THE FAGESTROM TEST**	

ALCOHOL

Have you ever had an entire drink?	Yes I No
Age when you had an entire drink for the first time	Numeral
Age when you have drunk an entire drink at least monthly for six months in a row	Numeral
Already drunk?	Yes I No I Never drank
How old was?	Numeral
How many times have you been drinking in the last year?	Numeral
Drinked at least once five drinks a day for two weeks	Yes I No I Never drank
How old was?	Numeral
Have you ingested a whole drink in the last 12 months?	Yes I No I Never drank
**APPLICATION OF THE AUDIT TEST**	
Already needed specific treatment for alcohol	Yes I No I Never drank
How old was?	Numeral
Type of treatment	Hospitalization I Outpatient clinic
Participated AA Groups	Yes I No I Never drank
How old was?	Numeral

CANNABIS

Have you ever used cannabis in your life?	Yes I No
Age when first used cannabis	Numeral
Age when consumed more cannabis	Numeral
Cannabis form that uses or uses more	Marijuana I Haxixe
Ever wanted to stop using cannabis?	Yes I No
Ever tried to stop using it seriously?	Yes I No

TRANQUILIZERS

Have you ever used tranquilizers in your life?	Yes I No I don’t now to say
Age when you first used tranquilizers	Numeral
Under what circumstances did it take tranquilizers for the first time	Doctor prescribed I Family gave me I A friend gave me I Anyone gave me I Nobody gave me I got myself
Why did you use?	Medical reason prescribed by a doctor I Medical reasons, by others I Non-medical reason for improving physical or cognitive performance I Non-medical, recreational reason
Age when most consumed tranquilizers	Numeral

AMPHETAMINES

Have you ever used amphetamines in your life?	Yes I No
Why did you use amphetamines?	To lose weight I To work without getting tired I For fun
During the phase in which he most used, he had the false sensation of being watched or persecuted?	Yes I No

ECSTASY

Have you ever used ectasy in your life?	Yes I No

HALLUCINOGENS

Have you ever used alucinogen in your life?	Yes I No

SOLVENTS

Have you ever used solvents in your life?	Yes I No

OPIUM DERIVATIVES

Have you ever used opiates in your life?	Yes I No

INVOLVEMENT IN ILLEGAL ACTIVITIES

Were you ever arrested before you were 18?	Yes I No
How old were you?	Numeral
How many times have you been in prison ?	one I two I three I four I more than 5
How old were you when you were last arrested?	Numeral
Have you had problems with misdemeanors and been approached by the police ever after the age of 18?	Yes I No
Why?	Porting of drugs I Fights or breaches of public order after use of alcohol and drugs I Selling or manufacturing drugs I Counterfeiting, fraud, receipt of stolen goods I Invasion of property, car theft and burglary I Thefts - "hitting a wallet", opening and removing objects from cars, picking up small objects in secret I Prostitution or other forms of sexual exploration I Robbery - banks, armed robbery I Homicide or guilty I Others
Did you use any drugs while you were in prison?	Yes I No

Have you ever sustained yourself for at least a month with illicit activities?	Yes l No
The most used activity for the sustenance	drug trafficking l betting game l thefts l robberies or robberies l forgery, fraud, embezzlement l prostitution or pimping
When was the last time you sustained like that?	1 month or less before admission l within the last 12 months l more than 12 months ago
In the last 30 days have you been involved in any illegal activity to get money to buy drugs?	Yes l No
What kind of illegal activity?	Selling or manufacturing drugs l Fake, fraud l Invasion of property , car theft l Thefts l Prostitution, pimping l Armed robbery l Kidnapping l Homicide l Others

SECTION GAINS AND SUSTAINABILITY

**In the last month before admission, where did the money come from? Type of gain:**	
Any form of legally recognized work, whether formal, informal or autonomous - If you are "removed by the INSS" - do not mark gains here.	Yes l No
Wage supplement - Program: “family bag”, ”food bag ...	Yes l No
Social security - retirement, unemployment benefits, sick leave	Yes l No
Personal income - applications, leases ...	Yes l No
Pension by post-separation agreement	Yes l No

"Allowance" or amounts received from third parties - spouse, parents, children, friends	Yes I No
Illegal activities - gambling, drug trafficking, robberies ...	Yes I No
Other sources. Which are?	Alms I Sale of personal belongings I Prostitution I Other

RELIGIOUSITY

You believe in God?	I believe, I am sure that God exists I do not believe, I'm sure God does not exist I do not know, I'm not sure if God exists
Do you have a religion?	Yes I No
What?	Catholic I Evangelicals I Spiritist Kardecista + Afro-Brazilian I Other
**Religious practice | Duke Religion Index**	
How often do you go to a church, temple, or other religious gathering?	More than once a week I Once a week I Two to three times a month I A few times a year I Once a year or less I Never
How often do you dedicate your time to individual religious activities, such as praying, praying, meditating, reading the Bible or other religious texts?	More than once a week I Once a week I Two to three times a month I A few times a year I Once a year or less I Never
"In my life, I feel the presence of Desus (or the Holy Spirit)." This statement is:	Totally Truth to Me I In general it is true I Not sure I In general it is not true I Not true
"My religious beliefs are really behind my whole way of living"	Totally Truth to Me I In general it is true I Not sure I In general it is not true I Not true
"I strive hard to live my religion in all aspects of life"	Totally Truth to Me I In general it is true I Not sure I In general it is not true I Not true

SEXUAL BEHAVIOR

How old were you when you had the first sexual intercourse?	Numeral
How important is sex to you?	Sex is important to my life I can live very well without it I never had
Are you satisfied with your sex life?	Yes I No
What is your sexual orientation, ie, what is your gender - man, woman, transvestite that attracts you the most?	I have no attraction for either sex I Heterosexual I Homosexual I Bisexual I Pansexual - includes attraction for transgender (transvestites)
Do you have one or more of a regular partner?	Yes I No
How many partners do you have?	Numeral
What kind of them?	Only men I Only women I Men and women I Men and shemales I Women and transvestites I Men, women and transvestites
Have you ever had sex with more than 10 different people in a single year?	Yes I No
Have you ever had sex with women in your life?	Yes I No
In the past 12 months, have you had any relation to how many different women?	I have not had I 1 women I Women I Women I 10-20 women I Women I 100 or more women
As for condom use, you:	Always uses I Sometimes uses I Usually uses I Never use
Do you know if any of the women you have sex with are injecting drug users or have had sex in exchange for drugs?	I Know That Yes I I'm sure you will not I I could not say

Have you ever had sex with men in your life?	Yes I No
In the past 12 months, have you had a relationship with how many different men?	I have not had I 1 man I Men I Men I 10-20 men I 21-99 men I 100 or more men
As for condom use, you:	Always uses I Sometimes uses I Usually uses I Never uses
Do you know if any of the men you have sex with are injecting drug users or have had sex in exchange for drugs?	I know I do I I'm sure it will not I could not tell
**What kind of sex do you practice?**	
Only sex without penetration (oral I masturbation)	Yes I No
Sex with vaginal penetration only	Yes I No
Sex with anal penetration only	Yes I No
Have you ever received drugs in exchange for sex?	Yes I No
Have you ever received drugs in exchange for sex in the past 12 months?	Never I Rarely I Sometimes I Frequently
Have you ever received drugs for sex in the past 12 months and have you ever used condoms?	I have always used I Few times I I have never used
In the past 12 months, have you paid, given gifts or provided favors in exchange for sex?	Yes I No
In the past 12 months, how many times have you paid, given gifts or provided favors in exchange for sex?	Rarely I Sometimes I Frequently

In the past 12 months, how many times have you used a condom when you paid, given gifts or provided favors in exchange for sex?	I've always used I Few times I Never used
In the last 12 months, have you ever received money, gifts or favors in exchange for sex?	Yes I No
In the past 12 months, how many times have you ever received money, gifts or favors in exchange for sex?	Rarely I Sometimes I Often
In the last 12 months, how many times have you used a condom when you ever received money, gifts or favors in exchange for sex?	I've always used I Few times I I have never used
Do you think you have any sexual dysfunction ("sexual problem")?	Yes I No
What dysfunction?	Desire decreased I Sexual Aversion I Difficulty in excitation or lubrication I Absence of orgasms I Pains in sexual intercourse I Vaginismus I Difficulty in erection or impotence I Premature Ejaculation I Delayed ejaculation I Other I Excessive Compulsion / Desire
Do you consider yourself dependent on sex?	Yes I No
